# Spatiotemporal dynamics of renal distal convoluted tubule dilatation and cyst formation in nephronophthisis type 1 mice

**DOI:** 10.1080/0886022X.2026.2684840

**Published:** 2026-06-23

**Authors:** Weina Zhang, Guanghua Xu, Xinyu Su, Jiayong Lai, Zhihe Xue, Tian Li, Hongbo Huang, Ruxin Li, Liangzhong Sun, Yanbing Li

**Affiliations:** ^a^ Department of Pediatrics, Nanfang Hospital, Southern Medical University, Guangzhou, China; ^b^ National Key Discipline of Human Anatomy, School of Basic Medical Sciences, Southern Medical University, Guangzhou, China

**Keywords:** Nephronophthisis, *NPHP*1, renal cyst, spatiotemporal dynamic, pathological anatomy

## Abstract

Nephronophthisis (NPH), a rare but serious ciliopathy, is the predominant genetic cause of end-stage kidney disease (ESKD) in children and adolescents. Renal tubular dilatation and cyst formation are key pathological features of NPH. The mechanisms underlying renal cystogenesis remain poorly understood. This study employed sex-balanced *Nphp1* deletion gene knockout (*Nphp1*^KO^) mice at 8, 12, and 24 weeks of age to investigate the spatiotemporal dynamics of renal tubules and cyst development, aiming to identify the origins of renal cysts. Quantitative evaluation of cyst progression revealed a significant increase in cyst number from 8 to 12 weeks of age, but no substantial change from 12 to 24 weeks. Additionally, cyst size remained stable throughout the study period. Serial section tracing and segment-specific tubular marker analysis confirmed that these cysts originated from the distal convoluted tubule (DCT), which displayed segmental dilation while preserving anatomical connections with adjacent, morphologically normal DCT. 3D reconstruction revealed that renal cysts appeared either as a single or in a bead-like arrangement. We demonstrated that human renal cyst epithelial cells from patients with NPH1 also originate primarily from the DCT. This study provides critical experimental evidence of the histopathological features of renal cysts in NPH1, advancing our understanding of the pathogenesis of NPHP-associated tubular dilation and cystogenesis.

## Graphical Abstract

**Figure:**
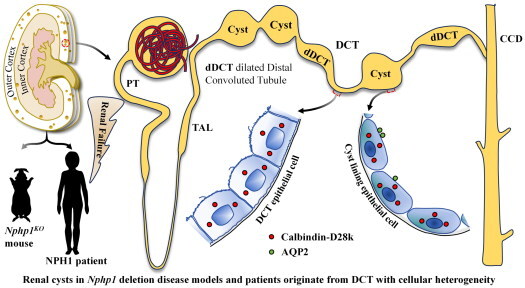

## Introduction

Nephronophthisis (NPH) is a prevalent monogenic cause of end-stage kidney disease (ESKD) in children and adolescents [1–3], constituting approximately 10% of ESKD cases in this age group [4]. The epidemiology of NPH has reported regional differences, with incidence rates of 1/50,000 and 1/100,000 in Canadian and American populations, respectively. NPH manifests in three subtypes based on the age of ESKD onset: infantile, juvenile, and adolescent/adult. The juvenile subtype predominates clinically, typically culminating in ESKD at a median age of 13.5 years [4]. While renal manifestations are primary in NPH, extrarenal features involving the liver, retina, and nervous system may occur [5]. Next-generation sequencing (NGS) has facilitated the identification of genes related to cystic kidney diseases, and studies have identified over 25 gene variants linked to NPH [6–8]. Among these, large homozygous deletions in the *NPHP*1 gene represent the predominant genetic mutation in juvenile NPH, constituting approximately 85% of cases with *NPHP*1 variants [9].

The pathogenesis of NPH remains incompletely understood, and effective targeted therapies are currently lacking. Kidney transplantation is considered a final resort. Initial clinical presentations of NPH are insidious [10], with most patients already displaying varying degrees of renal insufficiency upon diagnosis [11–12]. Clinical pathological studies of NPH are limited since renal biopsy specimens are scarce especially in the early stages of disease. Tubular dilation and the formation of renal cysts are prominent pathological features of NPH. Renal cysts in NPH are commonly located at the corticomedullary junction [2]. Nevertheless, the renal pathological characteristics associated with different genotypes of NPH exhibit variations [13–14]. Biopsies of infantile NPH patients with *NPHP2*, *NPHP3*, or *NPHP6* variants show microcysts in glomeruli with dilated Bowman's capsules [2,15]. A study utilizing a neonatal (inv/inv) mouse model resembling infantile NPH demonstrated uniform and diffuse cystic dilation in the collecting duct (CD) spanning from the medulla to the cortex, involving the CD, proximal tubule (PT), and ascending limbs of the loop of Henle [16]. Subsequently, in (pcy/pcy) mice with renal cysts due to *Nphp3* variants, segmental dilation mainly occurs in the CD and distal convoluted tubule (DCT), extending to all tubular segments in the later stages. With the progression of fibrosis, the proximal tubules atrophy [17]. Our group successfully constructed the first *Nphp1* deletion gene knockout mouse model (*Nphp1*^KO^) that mimics the renal phenotype of human type I NPH (NPH1) [18]. The spatiotemporal characteristics of renal cysts in this model have not been fully characterized. In-depth research on the characteristics of renal tubules and cysts in *Nphp1*^KO^ mice will contribute to clarifying the location, growth characteristics, and molecular features of the onset of NPH1 cysts.

This study examined renal tubular dilation and cyst formation in *Nphp1*^KO^ mice at 8, 12, and 24 weeks of age. The analysis revealed that renal cysts in these mice were small, predominantly located in the renal cortex and the corticomedullary junction area, and increased significantly in number between 8 and 12 weeks but not significantly between 12 and 24 weeks. However, cyst size did not show a significant increase with age. Additionally, the DCT in these mice exhibited segmental dilation at varying degrees at all three age points, forming a continuous structure with morphologically normal tubules. The cyst epithelial cells within dilated renal tubules consistently express distinctive markers of the DCT. Immunohistochemical analysis of serial sections confirms the DCT origin of these cyst epithelial cells. These cells demonstrate differentiation heterogeneity by co-expressing Calbindin-D28k and AQP2. This study's findings offer experimental support for elucidating the pathological processes underlying renal cyst formation in NPH and lay a foundation for targeted therapeutic investigations focusing on DCT lesions in this disease model.

## Materials and methods

### Mice

The *Nphp1*^KO^ mice were obtained from the laboratory of Professor LZ Sun. The research team previously utilized CRISPR/Cas9 gene editing to target and delete exons 2–20 of the *Nphp1* gene, successfully establishing the first C57BL/6J mouse model with human NPH renal phenotypes, including renal tubular dilation, and cyst formation [18]. A breeding colony of these mice was subsequently established at the Experimental Animal Research Center of Southern Medical University (12 L:12D cycle with *ad libitum* feeding). All animal experiments adhered to the ARRIVE guidelines (Supplementary Material, Checklist S1) and were conducted according to the protocol approved by the IACUC of Nanfang Hospital, Southern Medical University (Approval Number: IACUC-LAC-20220606-010). Genomic DNA was extracted from tail tissues of 3-week-old mice for genotyping C57BL/6J mice using a PCR-based method. The genotyping utilized the following primers: Nphp1-WT-F (5′-CTG GTC TAC CAT GAC TGC GTG-3′) and Nphp1-WT-R (5′-GCT CCA CTC TTC AAT GTG GGA-3′) for wild-type mice, and Nphp1-KO-F (5′-TAC ACA GTC GTG CCA GCC AG-3′) and Nphp1-KO-R (5′-GCT CCA CTC TTC AAT GTG GGA-3′) for knockout mice. Subsequent electrophoresis analysis categorized the mice into *Nphp1^+/+^* (wild-type, WT), *Nphp*1^+/−^ (heterozygous), and *Nphp1*^−/−^ (*Nphp1*^KO^) mice based on the PCR results.

### Human kidney tissue collection

Renal biopsy tissues were acquired from three pediatric NPH1 patients and three controls. The NPH1 patients (ages 10, 13, and 13) were genetically characterized by homozygous large deletions in the *NPHP*1 gene. The controls, comprising two 11-year-old males and one 13-year-old female, were diagnosed with frequent relapse primary nephrotic syndrome, and pathological examination revealed minimal change disease. This study was approved by the Ethics Committee of Nanfang Hospital, Southern Medical University (NFEC-2019-047). Prior to enrollment, written informed consent was obtained from the parents or legal guardians of all children participating in the study.

### Experimental design

A time-course study was conducted to assess the spatiotemporal morphological changes of renal tubules and renal cysts, along with cell proliferation. Sample sizes were determined based on the 3Rs principle to minimize animal use while ensuring statistical power. Mice were weighed weekly and sacrificed at 8, 12, and 24 weeks with a sample size of *n* = 3 males and *n* = 3 females per time point, and compared to age-matched WT mice (*n* = 3 males and *n* = 3 females per time point), totaling 36 mice. Mice were anesthetized with a 3% sodium pentobarbital intraperitoneal injection and euthanized. Body weight was recorded, and kidneys were collected and weighed. One kidney per mouse was randomly chosen and cut in half along the midline. The tissue was then fixed in 10% neutral buffered formalin for 24 h and later embedded in paraffin with the coronal surface upwards. The other kidney was also halved, with one part stored for protein extraction and the other for RNA extraction.

All outcome assessments were performed by two blinded pediatric nephrologists.

### RNA extraction and real-time quantitative PCR (qPCR)

Total RNA from mouse kidney was extracted using the RNA Isolator reagent (Vazyme), followed by cDNA synthesis using the HiScript II Q RT SuperMix kit (Vazyme). qPCR analysis was conducted using the ChamQ SYBR qPCR Master Mix (Vazyme) on a QuantStudio^™^ 5 system (Thermo Fisher). Relative mRNA expression was determined using the 2^−ΔΔCT method with Gapdh as the internal reference gene.

### Protein extraction and Western blotting

Tissue lysates were prepared using RIPA Lysis Buffer (Beyotime) supplemented with 1% PMSF (Fudebio) and 1% phosphatase inhibitor cocktail (Solarbio). After sonication and centrifugation (12,000 ×, 15 min, 4 °C), supernatants were collected and denatured at 100 °C for 10 min. Proteins were separated by SDS-PAGE and transferred to PVDF membranes. Immunoblotting was performed with primary antibodies against NPHP1 (1:1000; Sigma-Aldrich, SAB2104055) and GAPDH (1:1000; Fudebio, FD0063), followed by HRP-conjugated secondary antibodies. Protein bands were visualized using a Tanon imaging system (Tanon, China).

### Hematoxylin and eosin staining (H&E staining)

3-μm-thick sections were obtained from paraffin-embedded kidneys of *Nphp1*^KO^ mice, deparaffinized with xylene, and rehydrated through a graded ethanol series. H&E staining was performed using a commercial kit (HK20; Guangzhou Haoke Biotechnology Co., Ltd). Whole-slide images were acquired with a slide scanning system SQS-40R (TEKSQRAY, China). Cysts were defined as structures demonstrating a luminal diameter greater than 50 μm, based on previous literature standards [19]. Renal cyst diameters were measured in *Nphp1*^KO^ mice using ImageJ software by averaging the major and minor axes. The mean cyst diameter in 24-week-old *Nphp1*^KO^ mice was 63.05 ± 5.36 μm. To avoid duplicate sampling, three nonconsecutive sections (spaced 60 μm apart) from each kidney block were analyzed. H&E-stained sections were subjected to whole-slide scanning to evaluate the distribution of renal cysts within the renal parenchyma.

### Immunohistochemical and immunofluorescent staining

Kidney tissue sections (3 μm) from *Nphp1*^KO^ mice were deparaffinized and subjected to microwave-assisted antigen retrieval in EDTA buffer. Endogenous peroxidases were deactivated using 3% H_2_O_2_. Primary antibodies specific to different nephron segments were applied as follows: Calbindin-D28k (1:1000, Proteintech, 14479-1-AP) for DCT, AQP1 (1:6000, Proteintech, 20333-1-AP) for PT, AQP2 (1:500, Proteintech, 29386-1-AP) for cortical collecting ducts (CCD), and NKCC2 (1:2000, Proteintech, 189701-AP) for thick ascending limbs (TAL). Proliferation markers, PCNA (1:50, Santa Cruz, SC-56) and Cyclin D1 (1:100, Abcam, ab134175), were also utilized. Following incubation, slides were rinsed with PBS, treated with species-compatible HRP-secondary antibodies, and developed with DAB chromogen. Counterstaining with hematoxylin, ethanol dehydration, and neutral gum mounting were performed before whole-slide digital scanning (SQS-40R, TEKSQRAY) for histopathological evaluation. Human kidney sections were baked at 60 °C for 1 h and subsequently deparaffinized. Heat-induced antigen retrieval was then performed using Tris-EDTA buffer (pH 9.0; Servicebio). A TSA-plus based multiplex fluorescence staining kit (AiFang Biological, AFIHC024) was used for subsequent steps. Sections were incubated with primary antibodies against Calbindin-D28k (1:200, Proteintech, 14479-1-AP) and AQP2 (1:50, Proteintech, 29386-1-AP). All fluorescent images were acquired using a Nikon Eclipse Ti2-E inverted microscope.

### AI-based 3D reconstruction and visualization of distal convoluted tubules and renal cysts

Serial kidney sections (3 μm thick) from 24-week-old *Nphp1^KO^* mice were subjected to immunohistochemical staining for calbindin-D28k. Whole-slide images were acquired at 40× magnification using a Motic easyScan scanner (Xiamen, China). An artificial intelligence (AI) pipeline was employed to identify distal convoluted tubules (DCTs), dilated distal tubules, and renal cysts. The procedure consisted of the following steps: 1. Images were downsampled using a custom MATLAB script with nearest-neighbor interpolation. 2. Image registration was performed using an AI-based neural network to align all images with a reference. 3. Target structures were manually annotated on original images using ImageScope software, and the annotations were exported as XML files. 4. An AI network was trained on the annotated images and subsequently applied for semantic segmentation of the entire image set. 5. Following segmentation, a 3D reconstruction was generated to visualize tissue architecture. The segmentation masks were converted into a voxel grid using the ImageJ 3D Viewer plugin, and volume rendering was applied for final 3D visualization.

### Statistics

All experiments included three independent biological replicates. Data are expressed as mean ± standard deviation (SD). Image analysis was performed using ImageJ software, and positive cell quantification was conducted with QuPath (v5.1). Statistical analyses were executed in GraphPad Prism 10. For normally distributed data [1]: Inter-group comparisons used unpaired Student t-tests [2]; Multi-group comparisons used one-way ANOVA with Tukey *post hoc* testing. Nonnormally distributed data were analyzed by Mann-Whitney U tests. No animals or data points were excluded from the analysis. Statistical significance was defined at *p* < 0.05.

## Results

### Genotypic characterization and renal phenotype of Nphp1^KO^ mice

**Figure 1.**
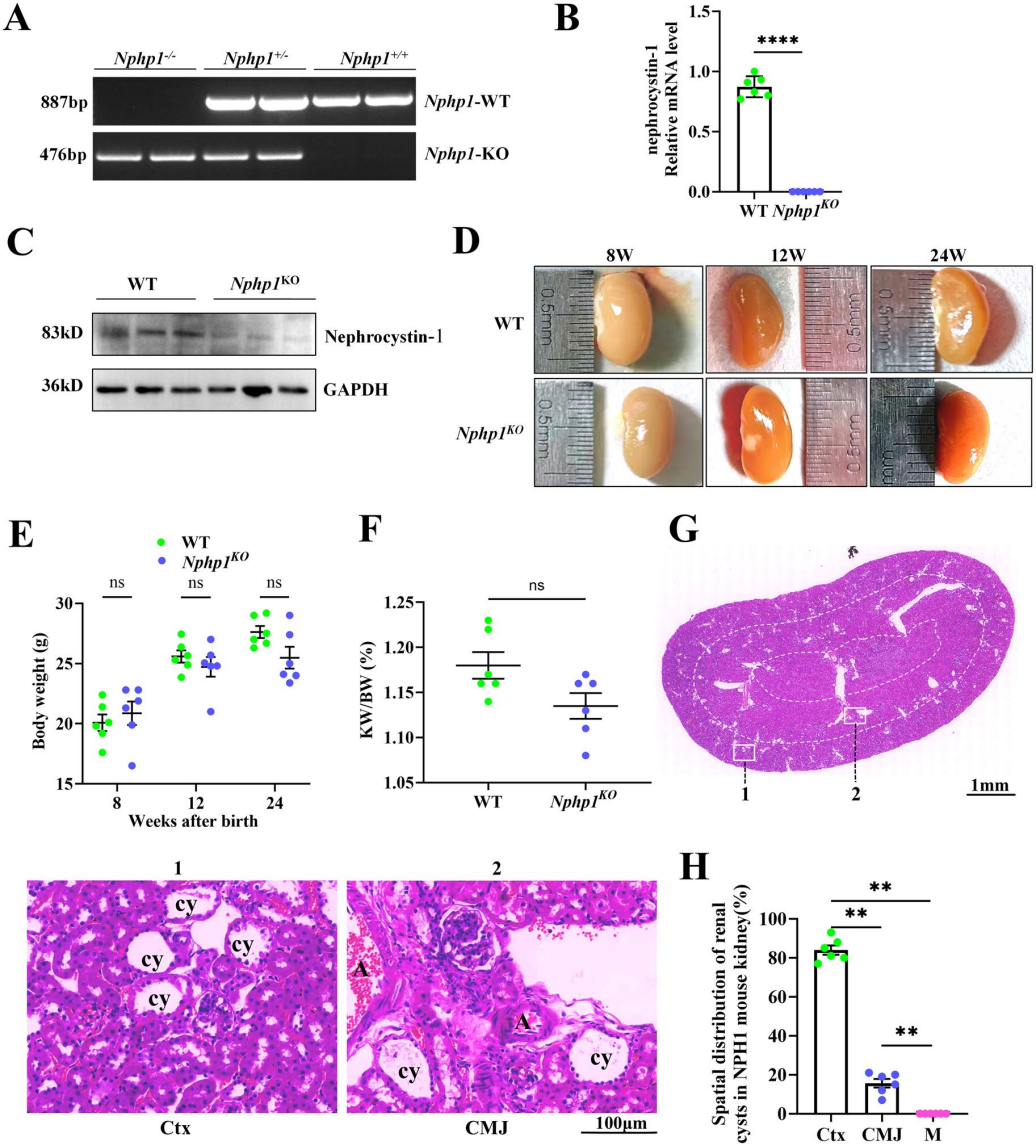
Genotype validation, molecular suppression, and renal phenotypes in *Nphp1*^KO^ mice. (A) Genotyping of 3-week-old mice by tail biopsy PCR. (B, C) Nephrocystin-1 mRNA and protein expression in kidney tissues. (D) Renal gross morphology of WT and *Nphp1*^KO^ mice at 8, 12, and 24 weeks. (E) Body weight of *Nphp1*^KO^ and WT littermates at indicated ages. (F) Kidney-to-body weight ratio (KW/BW) in 24-week-old mice. *n* = 6 per genotype. (G) H&E staining showing cyst distribution in renal cortex (Ctx) and corticomedullary junction area (CMJ) of an *Nphp1*^KO^ mice. (H) Quantification of cyst proportions in Ctx, CMJ, and medulla (M). (*n* = 6 mice; 3 sections/mouse). At low magnification, the CMJ is delineated by two white dotted lines. This region comprises the inner (juxtamedullary) cortex and the outer medulla. cy, cyst; A, artery. Data: mean ± SD. Significance: **p < 0.01; ns, not significant.

Genomic DNA was extracted from 3-week-old mice through tail clipping and then subjected to PCR amplification. Genotype determination was carried out using agarose gel electrophoresis. Mice were categorized into wild type (*Nphp1*^+/+^, WT), heterozygous type (*Nphp1*±), and homozygous knockout type (*Nphp1*^-/-^, *Nphp1*^KO^) based on the electrophoresis patterns of specific primers (Figure 1A, Fig. S1A). To validate the efficacy of *Nphp1* gene knockout, additional assessments were conducted using real-time quantitative PCR (qPCR) and Western Blot analyses. The findings revealed a significant reduction in *Nphp1* mRNA expression levels in kidney tissues of *Nphp1*^KO^ mice compared to those of wild-type controls (Figure 1(B)), with nearly complete absence of nephrocystin-1 expression (Figure 1(C), Figure S1B).

To investigate the phenotypic changes in the kidneys of *Nphp1*^KO^ mice during growth, we assessed the physical characteristics, body weight, and kidney dimensions (Figure 1(D)) of mice at different stages (8, 12, and 24 weeks) and compared them with age-matched WT mice. Our findings revealed that the physical appearance of *Nphp1*^KO^ mice resembled that of WT mice at corresponding ages, and no significant differences in body weight were observed (Figure 1(E)). By 24 weeks, there was no notable variance in the kidney weight-to-body weight ratio between *Nphp1*^KO^ and WT mice (Figure 1(F)). These results align with clinical observations of normal or diminished kidney size in individuals with type I NPH [20–22]. Our understanding of early renal pathology in type 1 NPH is limited due to the insidious progression of the disease. The *Nphp1*^KO^ mice serve as the initial mouse model replicating the kidney phenotype seen in human type 1 NPH. Examination of kidney tissue sections from 8-week-old *Nphp1*^KO^ mice revealed scattered cysts in the renal cortex. By the age of 12 and 24 weeks, renal cysts in *Nphp1*^KO^ mice increased, more located in the cortex but in smaller size, and a proportion of cysts in the corticomedullary junction area (CMJ) which includes the inner cortex and the outer medulla, while almost no cysts were found in the renal medulla (Figure 1(G)). The distribution of renal cysts among the cortex, CMJ, and medulla showed a significant difference (Figure 1(H)).

### Lack of progressive renal cyst expansion in Nphp1^KO^ mice with aging

**Figure 2.**
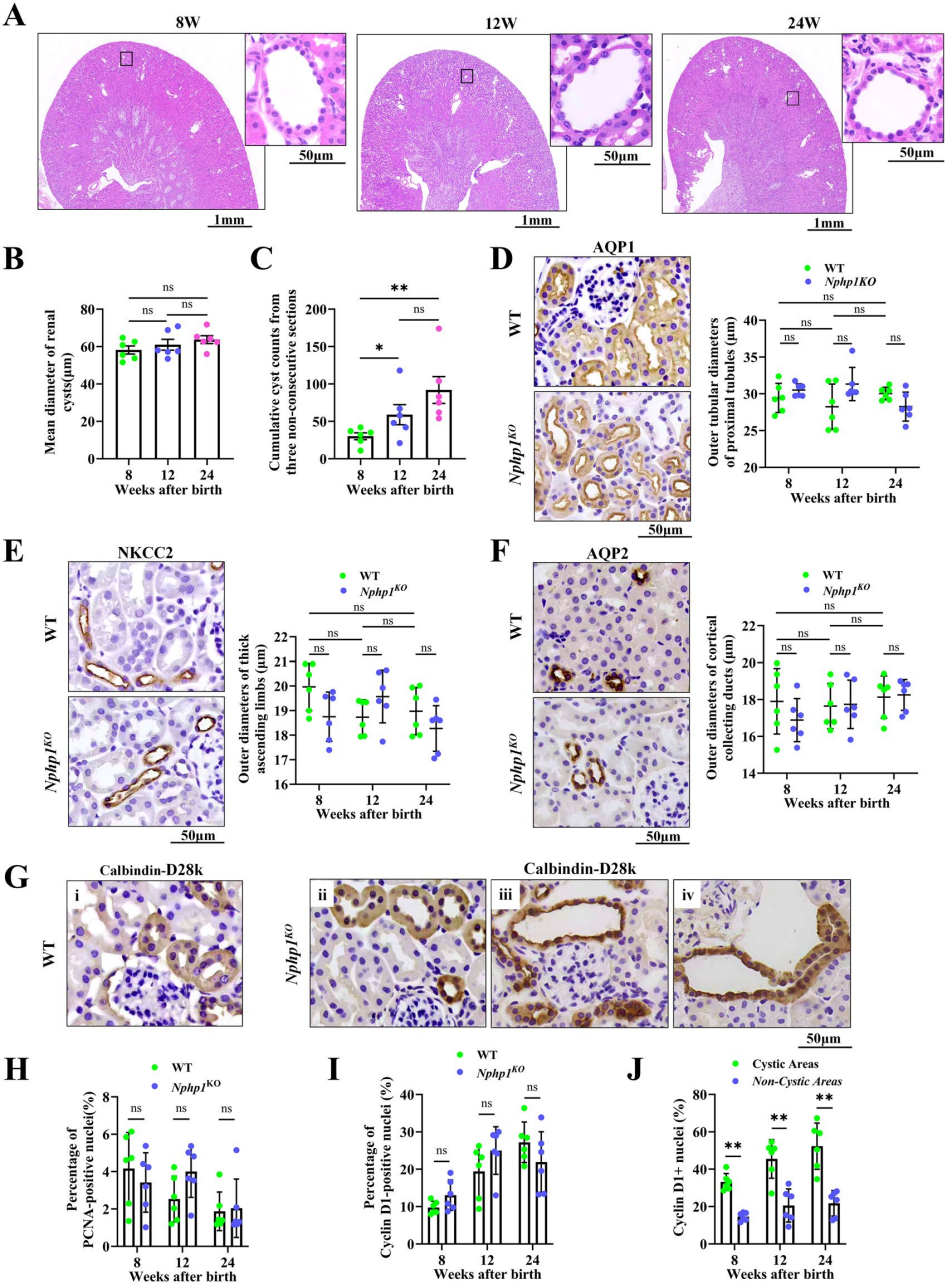
Progressive cystic and tubular pathology in *Nphp1*^KO^ mouse kidneys at 8, 12, and 24 weeks. (A) Age-dependent cystic changes in *Nphp1*^KO^ mice: Comprehensive low-magnification overviews (scale bar = 1 mm) and high-magnification cyst morphology (scale bar = 50 μm). (B) Developmental progression of renal cyst diameters in *Nphp1*^KO^ mice. (C) Quantification of renal cysts in *Nphp1*^KO^ mice, based on three nonconsecutive sections (60 μm intervals) per animal. (D) Proximal tubule morphology and outer diameter quantitative analysis in age-matched WT versus *Nphp1*^KO^ mice. (E) Thick ascending limb morphology and outer diameter analysis in WT versus *Nphp1*^KO^ mice. (F) Collecting duct morphology and luminal diameter analysis in WT versus *Nphp1*^KO^ mice. (G) Distal convoluted tubule morphology: WT (i) versus *Nphp1*^KO^ mice showing normal (ii) and segmentally dilated tubules (iii, iv). (H, I) Percentages of PCNA/Cyclin D1-positive cells in cortical fields from WT and *Nphp1*^KO^ renal sections. (J) Cyclin D1-positive cell percentages in cystic versus non-cystic regions of *Nphp1*^KO^ mice. (*n* = 6 mice/group; 3 images/mouse. Scale bars = 50 μm. Data: means ± SD. *p < 0.05, **p < 0.01; ns: not significant.).

Although renal cysts were not readily apparent in low-magnitude H&E-stained kidney sections from 8-, 12-, and 24-week-old *Nphp1*^KO^ mice, high-magnification imaging revealed uniformly small cysts (Figure 2(A)). Mean cyst diameters were 58.09 ± 4.96 μm (8 weeks), 61.04 ± 7.29 μm (12 weeks), and 63.05 ± 5.36 μm (24 weeks). Cyst size remained stable with aging, showing no significant increase over time (*p* > 0.05, Figure 2(B)). Cyst number significantly increased between 8 and 12 weeks (*p* < 0.05) but plateaued from 12 to 24 weeks (*p* > 0.05, Figure 2(C)).

## Focal tubular dilation in the DCT of *Nphp1*^KO^ mice

The DCT of *Nphp1*^KO^ mice exhibited segmental dilation. Using specific nephron segment markers (AQP1; AQP2; NKCC2 and Calbindin-D28k), we found no significant difference in the outer diameter of PT between 8-, 12-, and 24-week-old *Nphp1*^KO^ mice and age-matched WT mice (Figure 2D). Since renal cysts in *Nphp1*^KO^ mice were localized to the cortex, we separately quantified the outer diameters of CCD and TAL, comparing them with those of WT mice. Results showed no statistically significant differences in the outer diameters of CCD or TAL between *Nphp1*^KO^ and WT mice of the same age (Figure 2E, F). Furthermore, the PT, CCD, and TAL did not widen significantly in *Nphp1*^KO^ mice from 8 to 24 weeks of age, maintaining relatively constant outer diameters (Figure 2D–F). Notably, compared to DCT morphology in WT mice (Figure 2G, i), *Nphp1*^KO^ mice displayed both normal (Figure 2G, ii) and deformed DCT segments which manifested segmental dilation (Figure 2G, iii, iv). These dilated tubules—observed in mice aged 8, 12, or 24 weeks—featured enlarged lumens, thinner epithelium, sparse cytoplasm, and smaller epithelial nuclei compared to adjacent undilated DCT.

Our experimental results showed that the proliferative marker PCNA and Cyclin D1 displayed similar expression levels in the renal cortex of *Nphp1*^KO^ mice and age-matched WT controls, indicating no statistically significant difference (Figure 2(H,I)). This suggests that overall renal cellular proliferation is unaffected in *Nphp1*^KO^ mice. However, we observed region-specific dysregulation in Cyclin D1 expression: a notable increase was specifically observed within the cystic epithelium of *Nphp1*^KO^ renal cortex compared to neighboring non-cystic tubules (Figure 2(J)). This spatially varying pattern of Cyclin D1 expression may be associated with tubular dilation and subsequent cyst formation.

### Renal cyst epithelium primarily originates from DCT in Nphp1^KO^ mice

**Figure 3.**
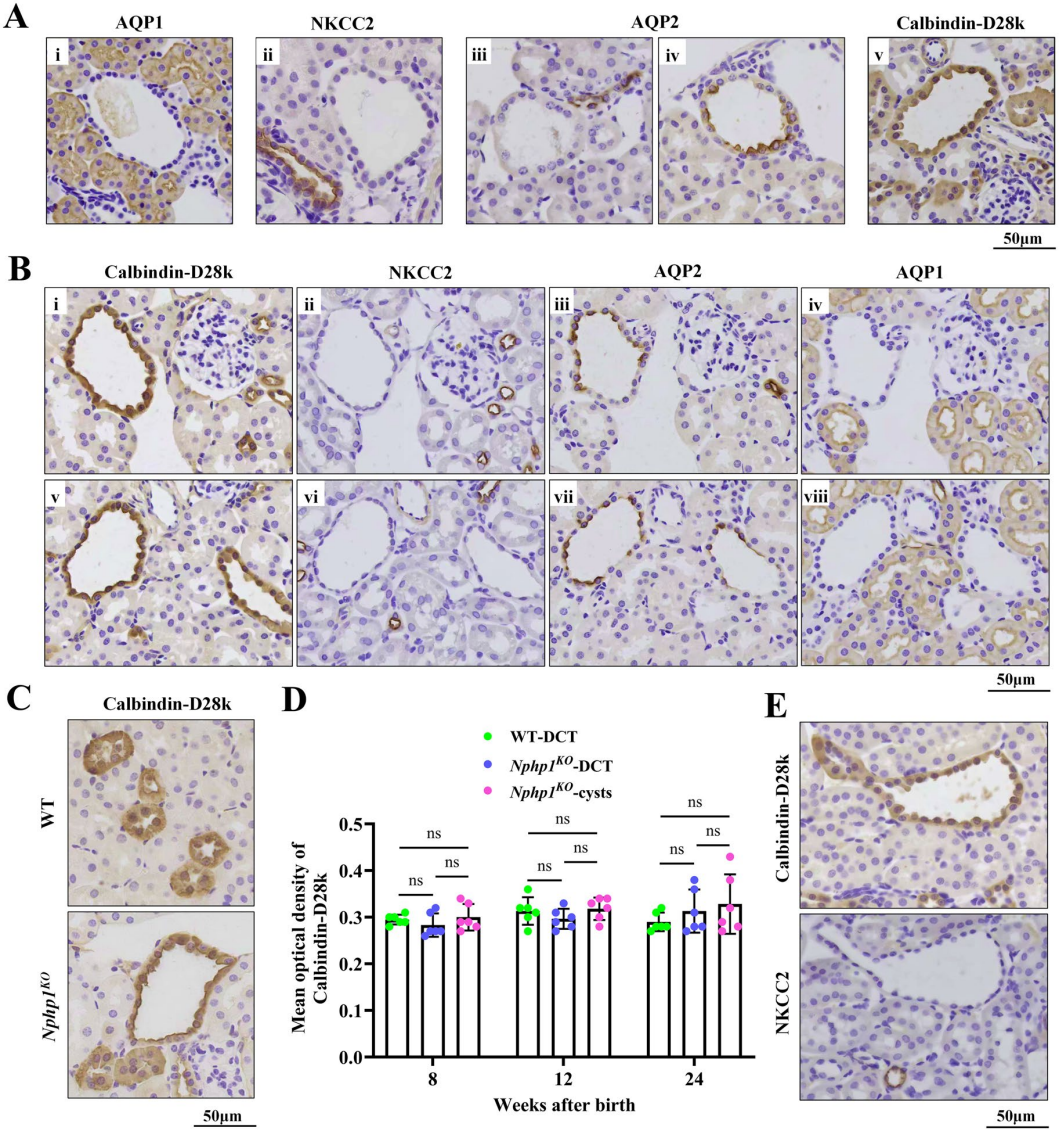
Cyst-lining epithelial cells originate from distal convoluted tubules in ***Nphp1*^KO^** mice. (A) Expression of nephron segment-specific markers, including AQP1 (i), NKCC2 (ii), AQP2 (iii, iv), and Calbindin-D28k (v), in cyst-lining epithelial cells of ***Nphp1*^KO^** mice. (B) Expression of nephron segment-specific markers in epithelial cells within the same renal cyst on consecutive tissue sections (i-iv: serial sections; v-vii: serial sections). (C) Expression of Calbindin-D28k in distal convoluted tubules of wild-type mice (WT-DCT), non-dilated distal convoluted tubules of ***Nphp1*^KO^** mice (***Nphp1*^KO^** -DCT), and renal cyst epithelia (***Nphp1*^KO^** - cysts). (D) Semi-quantitative comparison of Calbindin-D28k expression using mean optical density (MOD) in WT-DCT vs. ***Nphp1*^KO^** -DCT vs. ***Nphp1*^KO^** -cysts (*n* = 6 mice/group, scale bar = 50 μm. Data presented as means ± SDs. ns, not significant). (E) Serial sections showed the cyst connected to renal tubule.

Immunohistochemical analysis was conducted to investigate the origin of renal cyst epithelium in *Nphp1*^KO^ mice by assessing the expression of segment-specific nephron markers. AQP1 was consistently absent in renal cyst epithelial cells throughout the experimental period, in contrast to the positive staining observed in adjacent proximal convoluted tubules (Figure 3(A, i)). Similarly, NKCC2 was not detected in renal cyst epithelial cells (Figure 3(A, ii)). AQP2 displayed a varied expression pattern within renal cyst epithelial cells, ranging from near absence in some cysts (Figure 3(A, iii)) to discontinuous expression in others, with partial positivity in cyst epithelial cells and complete absence in adjacent regions (Figure 3(A, iv)). Calbindin-D28k consistently exhibited strong positive cytoplasmic expression in all cyst epithelial cells across kidney tissue sections from mice at all experimental time points (Figure 3(A, v)).

Serial sections of kidney tissue from *Nphp1*^KO^ mice were examined to elucidate the origin of cyst epithelium. 3D histology was utilized to track marker expression in renal tubular segments within the epithelial cells of cysts. Immunohistochemical analysis revealed distinct marker expression profiles in renal cyst epithelial cells of *Nphp1*^KO^ mice: AQP1 and NKCC2 were consistently negative, AQP2 showed intermittent expression, and Calbindin-D28k exhibited stable positivity (Figure 3B, i-iv and v-viii). This expression pattern remained consistent across the observed age range of 8-12-24 weeks. The Calbindin-D28k expression in cyst epithelium closely resembled that in the DCT of WT mice (Figure 3(C)). Semi-quantitative analysis confirmed no significant difference in the intensity of Calbindin-D28k expression between cysts, undilated DCT of *Nphp1*^KO^ mice, and DCT of WT mice (Figure 3(D)). Morphological examination indicated that the presence of Calbindin-D28k positive markers distinctly delineated the sequential pathological changes from a normal lumen to cystic dilatation (Figure 3(E)). These findings suggest that the deletion of *Nphp1* may selectively affect specific segments of the DCT, leading to progressive dilation and eventual cyst formation.

**Figure 4.**
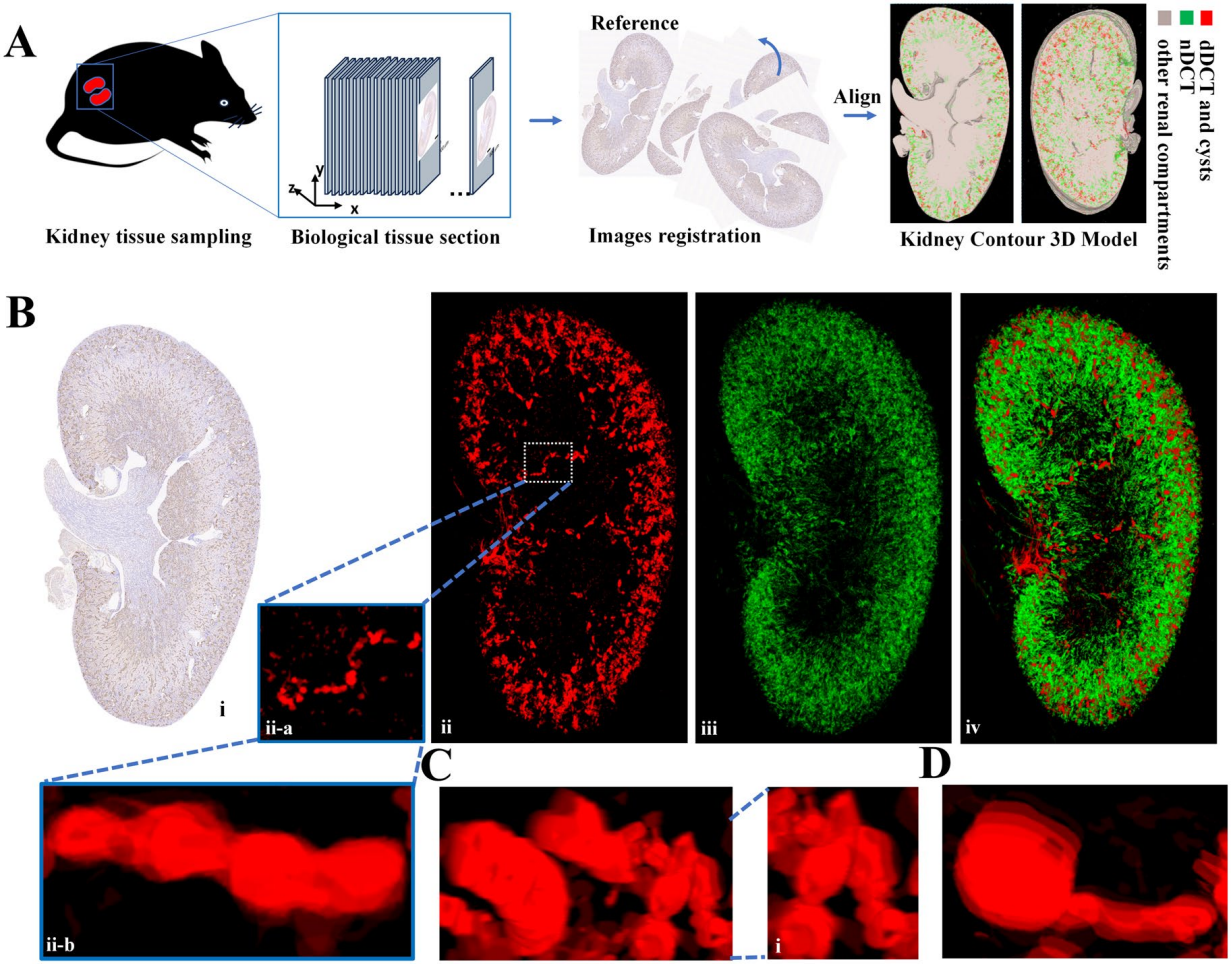
3D visualization of renal cysts in 24-week-old ***Nphp1*^KO^** mice. (A) Schematic workflow of the 3D reconstruction process and the resultant renal architecture model.(B) Representative images and 3D models derived from ***Nphp1*^KO^** mice kidney sections: (i) histological section of ***Nphp1*^KO^** mouse kidney tissue (immunohistochemical staining); (ii) 3D model of dilated distal convoluted tubules(dDCT) and renal cysts, with magnified views illustrating the bead-like morphology of the cysts (ii-a, ii-b); (iii) 3D model of normal distal convoluted tubules(nDCT); (iv) merged image of nDCT (green), dDCT and renal cysts (red); (C) Representative 3D model showing dDCT and renal cysts (i). (D) 3D reconstruction illustrating the connection between a renal cyst and the distal convoluted tubule.

To further characterize the cystic structures, we performed AI-based 3D reconstruction using sequential renal tissue sections from 24-week-old *Nphp1^KO^* mice (Figure 4(A)). Calbindin-D28k was used as a marker to identify DCT, dilated tubules, and cysts (Figure 4B, i). The 3D reconstruction results were consistent with two-dimensional histopathological observations, confirming that renal cysts in *Nphp1^KO^* mice were predominantly located in the outer cortex, with fewer detected in the inner cortex and corticomedullary junction (Figure 4(B)). Moreover, the reconstructions revealed that renal cysts not only occurred as single cyst connected to DCT (Figure 4(D)), but also frequently exhibited a distinctive beaded appearance (Figure 4(B), ii-a and ii-b; Figure 4(C)).

### Renal cyst epithelium in NPH1 patients originates primarily from the DCT

**Figure 5.**
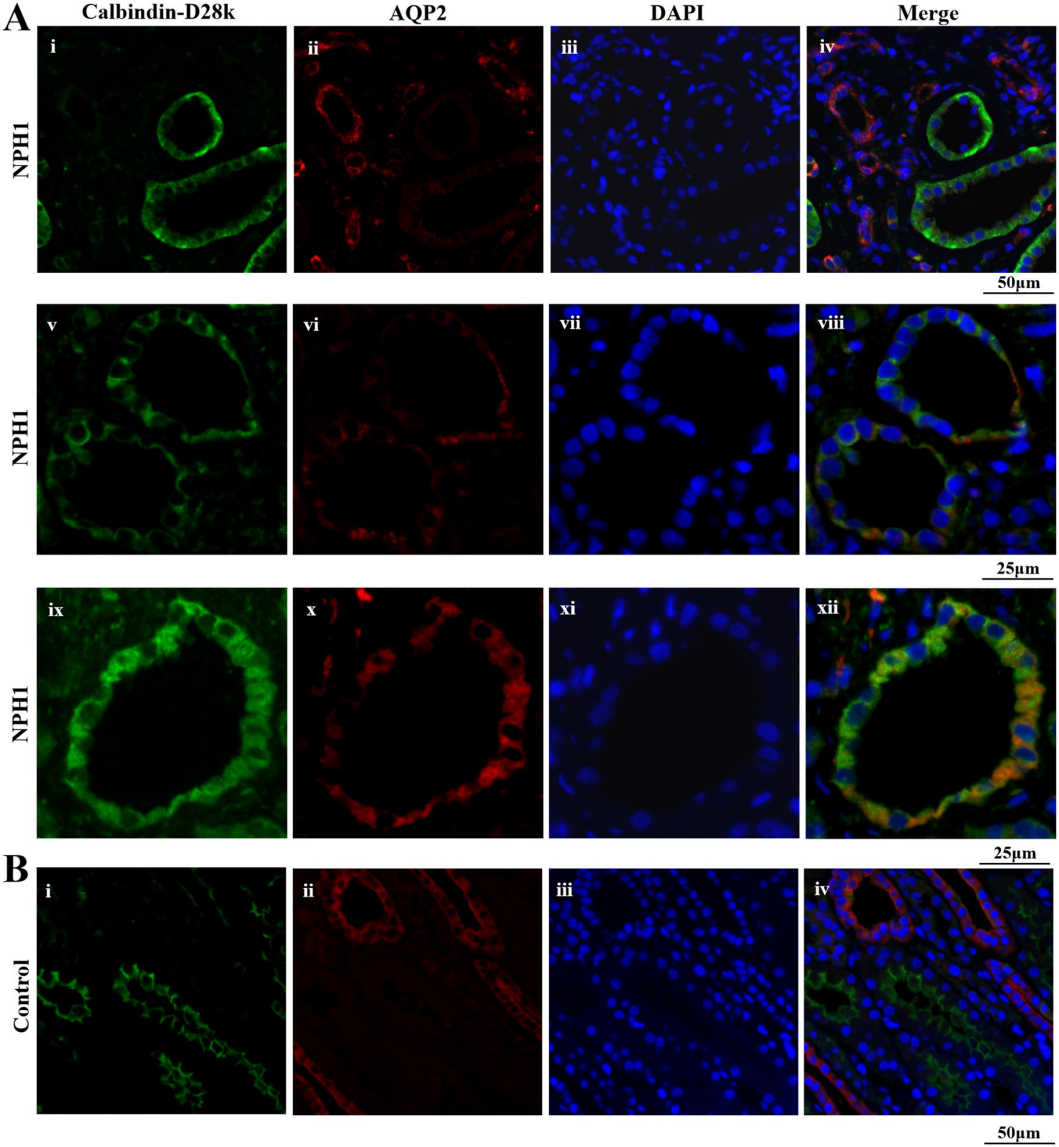
Representative immunofluorescence images of Calbindin-D28k and AQP2 in renal tissues from NPH1 patients and controls. (A) Expression of Calbindin-D28k (green) and AQP2 (red) in dilated tubular epithelial cells (i-iv; scale bar = 50 µm) and cystic cells (v-xii; scale bar = 25 µm) of NPH1 patients. (B) Expression of Calbindin-D28k (green) and AQP2 (red) in renal tissues from control subjects (i-iv; scale bar = 50 µm). Nuclei were stained with DAPI (blue).

To validate the cellular origin of renal cysts resulting from *NPHP1* deficiency, we performed immunofluorescence on NPH1 patient kidney tissues. The results demonstrated sustained and prominent expression of Calbindin-D28k in the epithelia of dilated tubules and cysts (Figure 5A, i, v and ix), consistent with our findings in *Nphp1*^KO^ mice. Moreover, the expression pattern of AQP2 in patient samples resembled that in *Nphp1*^KO^ mice: it was largely negative in dilated tubules but exhibited attenuated and discontinuous expression in the cyst linings (Figure 5A, ii, vi and x). In the control group, Calbindin-D28k was expressed in the epithelial cells of the DCT (Figure 5B, i), while AQP2 was expressed in the epithelial cells of the CCD (Figure 5B, ii). Collectively, these data indicate that the “negative or partially positive” expression pattern of AQP2, the characteristic observed in *Nphp1*^KO^ mice, is recapitulated in renal tissues from NPH1 patients.

## Discussion

Renal cysts are the common pathological basis of cystic kidney diseases and a shared factor contributing to renal insufficiency [17,23,24]. Hereditary cystic kidney diseases are primarily classified into: autosomal dominant polycystic kidney disease (ADPKD), autosomal recessive polycystic kidney disease (ARPKD), and nephronophthisis-related ciliopathies (NPHP-RCs) [25–26]. NPH1 is rare, with an insidious yet severe disease progression. Most clinicopathological diagnoses and studies rely on renal biopsy specimens from patients who already have renal insufficiency, while pathological studies of the early stages of the disease are scarce. The *Nphp1* deletion (*Nphp1*^KO^) mouse, as the first model to recapitulate the renal phenotype of human NPH, provides a reliable tool for investigating the pathological mechanisms of type 1 NPH. Our findings indicate that *Nphp1* deletion did not significantly affect mouse growth or development. The body weights of *Nphp1*^KO^ mice at 8, 12, and 24 weeks were not significantly different from those of age-matched WT mice. Additionally, at 24 weeks of age, kidney size in *Nphp1*^KO^ mice was not significantly different compared to WT mice. Prior research on *Nphp1*^KO^ mice suggests a gradual disease progression in this model, with renal dysfunction appearing only at 72 weeks of age [18]. In contrast to previously reported NPH models featuring deletions of apoptotic regulatory factors in renal tubular epithelial cells, which exhibit progressive weight loss, renal atrophy, and shortened lifespan [27], the *Nphp1*^KO^ mice more closely phenocopy human NPH1. We further analyzed the histological distribution of renal cysts in *Nphp1*^KO^ mice. In human NPH, cysts are typically located at the corticomedullary junction. However, in *Nphp1*^KO^ mice, cysts were distributed in both the renal cortex and the corticomedullary junction area, with more cysts in the cortical region, though smaller in size. Our previous observations in this model predominantly focused on cysts at the corticomedullary junction. In this study, however, we observed numerous dilated cortical tubules that had previously received little attention and that met the criteria for small cysts, which partially explains the increased cortical cyst count. Additionally, the slight difference in amino acid sequence of human and mouse Nphp1 proteins (91% similarity, 83% identity) [28] could also impact the differences in renal pathology between human NPH and the mouse model. This research performed the initial microanatomical mapping of renal cyst distribution in NPH1 model, providing essential histological understanding of cyst formation mechanisms.

Renal cystogenesis in cystic kidney disease involves multifaceted mechanisms, such as impaired planar cell polarity, defective ciliary function, aberrant cellular proliferation, and dysregulated signaling pathways [2,7,14,29]. Inherited cystic kidney diseases exhibit genotype-specific phenotypes [14]。ARPKD and NPH predominantly affect children and adolescents, with ARPKD often progressing rapidly. Conversely, ADPKD is less common in children, characterized by larger, more numerous cysts diffusely distributed in the renal parenchyma, resulting in significant kidney enlargement [30]。In NPH, cysts are typically smaller, and kidneys may appear normal or reduced in size [21,31]. Given the small size of renal cysts in *Nphp1*^KO^ mice, which precluded the use of the Cystic Index, this study quantified renal cyst burden at 8, 12, and 24 weeks of age using cyst counts and average diameter measurements. Analysis revealed a significant increase in cyst number between 8 and 12 weeks, with no substantial growth trend observed from 12 to 24 weeks. This temporal pattern indicates that peak cyst proliferation likely occurs by or before 12 weeks of age. Building on this study, future investigations targeting potential progenitor subpopulations could more rigorously explore the progenitor potential of this subset, allowing for a more detailed dissection of early cyst-initiating events. Notably, average cyst diameter remained consistent across all timepoints, suggesting potential underlying mechanisms may contribute to restricted cyst expansion and size maintenance. Furthermore, renal tissues from both *Nphp1*^KO^ mice and wild-type littermates exhibited comparably low PCNA expression without significant intergroup differences. However, Cyclin D1 expression differed significantly between cystic and non-cystic regions in *Nphp1*^KO^ kidneys, potentially linked to NPH-associated tubular dilation and cyst formation. Previous studies have shown that early cyst growth in ARPKD model rats (lpk strain) is linked to increased nuclear expression of Cyclin D1/Rb proteins [32]. Similarly, in the unilateral ureteral obstruction (UUO) model, elevated cyclin D1 expression correlates with renal tubular dilation and interstitial inflammation [33]. Thus, elevated Cyclin D1 expression in cystic regions of *Nphp1*^KO^ mice kidneys may contribute to cyst progression and/or renal interstitial inflammation, warranting further investigation to elucidate the underlying mechanisms. Collectively, cellular proliferation activity in renal tissues of *Nphp1*^KO^ mice persists at low levels, distinct from the aberrant proliferation characteristic of PKD [32,34]. In contrast to the numerous large renal cysts and significantly enlarged kidneys seen in PKD, the cysts in NPH are generally smaller and remain relatively stable. Studies suggest that the Hippo signaling pathway serves as a critical determinant between NPH and PKD in terms of cellular proliferation. This pathway regulates tissue and cellular growth and proliferation by modulating the subcellular localization (nuclear/cytoplasmic) and activity of the transcriptional coactivators YAP and TAZ [35]. In ADPKD, the Hippo signaling pathway is generally inactive, leading to excessive cell proliferation. Conversely, in *Nphp* gene mutation models mimicking human NPH (such as *Nphp4* or *Nphp1* deficiency), the Hippo signaling pathway is abnormally activated, persistently suppressing the transcriptional activity of YAP/TAZ and their pro-proliferative effects, ultimately restricting cellular proliferative capacity [36–37]. These mechanisms provide a molecular-level explanation for the characteristic manifestations of NPH, where patients typically exhibit normal or reduced kidney size, and cyst dimensions do not significantly expand with age.

Renal interstitial fibrosis is a key factor in the declining renal function associated with NPH, with tubular dilation and inflammatory cell infiltration serving as fundamental contributors to its development [38–39]. However, the temporal progression of these pathologies may vary by models. In the *Nphp1*^KO^ mouse, tubulointerstitial fibrosis is minimal at 12 weeks, more frequently emerges by 36 weeks, and is pronounced by 72 weeks. Thus, tubular dilatation and cyst development are early features of NPH1 in this model [18]. Recent 3D reconstruction studies of kidney tissue clearing in Jck mice have revealed that renal cyst development may be confined to specific tubular segments [40]. Immunohistochemical staining in our study showed no dilation in the outer diameters of the PT, TAL, and CCD in *Nphp1*^KO^ mice at 8, 12, and 24 weeks. Given that renal tubule maturation in normal mice is complete by 8 weeks [41], we infer that the PT, TAL, and CCD in *Nphp1*^KO^ mice retain developmental characteristics akin to those in WT mice throughout the study period. The findings suggest that the cysts resulting from *Nphp1* deficiency may originate from other renal tubular segments or specific cell types. Immunohistochemical analysis of renal tissue sections labeled with Calbindin-D28k revealed significant morphological heterogeneity in the DCT of *Nphp1*^KO^ mice. While some segmental DCT maintained normal morphology, others exhibited varying degrees of dilation, characterized by flattened epithelial cells, increased luminal and external diameters, and a segmental distribution of dilation that contrasted with adjacent normally shaped tubules. These observations indicate that *Nphp1* deletion may lead to selective segmental dilation of DCT, ultimately resulting in cyst-like structures.

Serial section immunohistochemical studies on renal tissues from *Nphp1*^KO^ mice were conducted to validate the hypothesis. The experimental results demonstrated that within the same cyst, the PT marker AQP1 and the TAL marker NKCC2 were both negatively expressed in the cyst-lining epithelium, while the CCD marker AQP2 showed negative or discontinuous positive expression, suggesting incomplete differentiation of the cyst epithelium toward a collecting duct phenotype. Notably, the DCT marker Calbindin-D28k was consistently expressed in the cytoplasm of cyst epithelial cells, and its semi-quantitative expression levels (based on mean optical density) showed no significant differences compared to non-dilated distal convoluted tubules (DCTs) in *Nphp1*^KO^ mice or DCTs in WT mice. These characteristics observed in serial tissue sections suggest that the cyst epithelium may originate from early DCT segments and may undergo abnormal differentiation during cyst formation. This finding aligns with single-nucleus RNA sequencing (snRNA-seq) data from renal tissues of 12-week-old *Nphp1*^KO^ mice. The snRNA-seq analysis indicates that NPH1-associated cystic cells may originate from the DCT3 cell subpopulation. This group is located in the initial region of renal tubule differentiation and has unique molecular characteristics: unlike DCT1 cells, which express typical distal convoluted tubule markers, DCT3 cells show reduced expression of these markers and aberrantly express some principal cell markers of the collecting duct [42]. Further Gene Ontology (GO) enrichment analysis of differentially expressed genes revealed significant downregulation of genes involved in renal tubule development and morphogenesis in DCT3 cells [42]. We show that renal cysts in NPH1 patients stem from DCT but display cellular heterogeneity, indicated by partial AQP2 expression. This observation implies that these cysts could originate from an early DCT precursor cell that maintains the ability to differentiate in both DCT and CD lineages. Additionally, histological examination of kidney sections from *Nphp1*^KO^ mice at various ages showed that cystic structures remain anatomically continuous with adjacent non-dilated DCTs. Notably, our 3D reconstructions revealed that renal cysts in this model presented either as single structure or in a beaded morphology, frequently continuous with tubular segments. This finding provides direct morphological evidence for cyst origin: 1) spatially, it confirms that cysts arise from the epithelium of the distal convoluted tubule; 2) it indicates that cyst formation may occur through localized tubular dilation rather than complete detachment. This is a notable contrast to ADPKD, where cysts frequently detach entirely from the original tubular structure [43]. Such structural differences suggest distinct pathogenic mechanisms: NPH likely results from segmental tubular dilation due to altered epithelial cell polarity, whereas ADPKD involves extensive cyst wall remodeling and independent growth.

In the *Nphp1*^KO^ mice model, renal cysts were predominantly localized to the renal cortex, exhibiting relatively small size and stable dimensions. Histological analysis revealed segmental dilation of the DCT, with both dilated tubules and cysts maintaining anatomical continuity with adjacent morphologically normal distal tubules. The current study was limited to the analysis of *Nphp1*^KO^ mice at specific ages. Additionally, the small sample size available for clinical validation, due to the rarity of the disease and the distinct characteristics of the pediatric population, warrants further validation. The underlying molecular mechanisms driving the segmental dilation of the distal convoluted tubule and subsequent cyst initiation remain an critical area for future investigation.

## Conclusions

Integrating the experimental results of this study with previous findings from Professor LZ Sun's group [21,42], we conclude that the renal cyst epithelial cells in NPH1 may originate from an early-stage, differentiationally heterogeneous subpopulation of distal tubule cells. This cell population expresses distal tubule-specific marker and simultaneously in some cells express molecular marker typically enriched in collecting duct epithelial cells. NPH1 renal cyst development may be confined to the DCT segments. This study offers critical experimental insights into the mechanisms of DCT lesions and the exploration of targeted therapeutic strategies.

## Supplementary Material

Fig S1.tif

table S1B.xlsx

table S1A.xlsx

## Data Availability

The data that support the findings of this study are available from the corresponding author, [Y.L.], upon reasonable request.
