# Supplementary figures and images for: Spatiotemporal dynamics of renal distal convoluted tubule dilatation and cyst formation in nephronophthisis type 1 mice
Source: Ren Fail. 2026 Jun 23;48(1):2684840. doi: 10.1080/0886022X.2026.2684840 (PMC13292302; doi:10.1080/0886022X.2026.2684840)

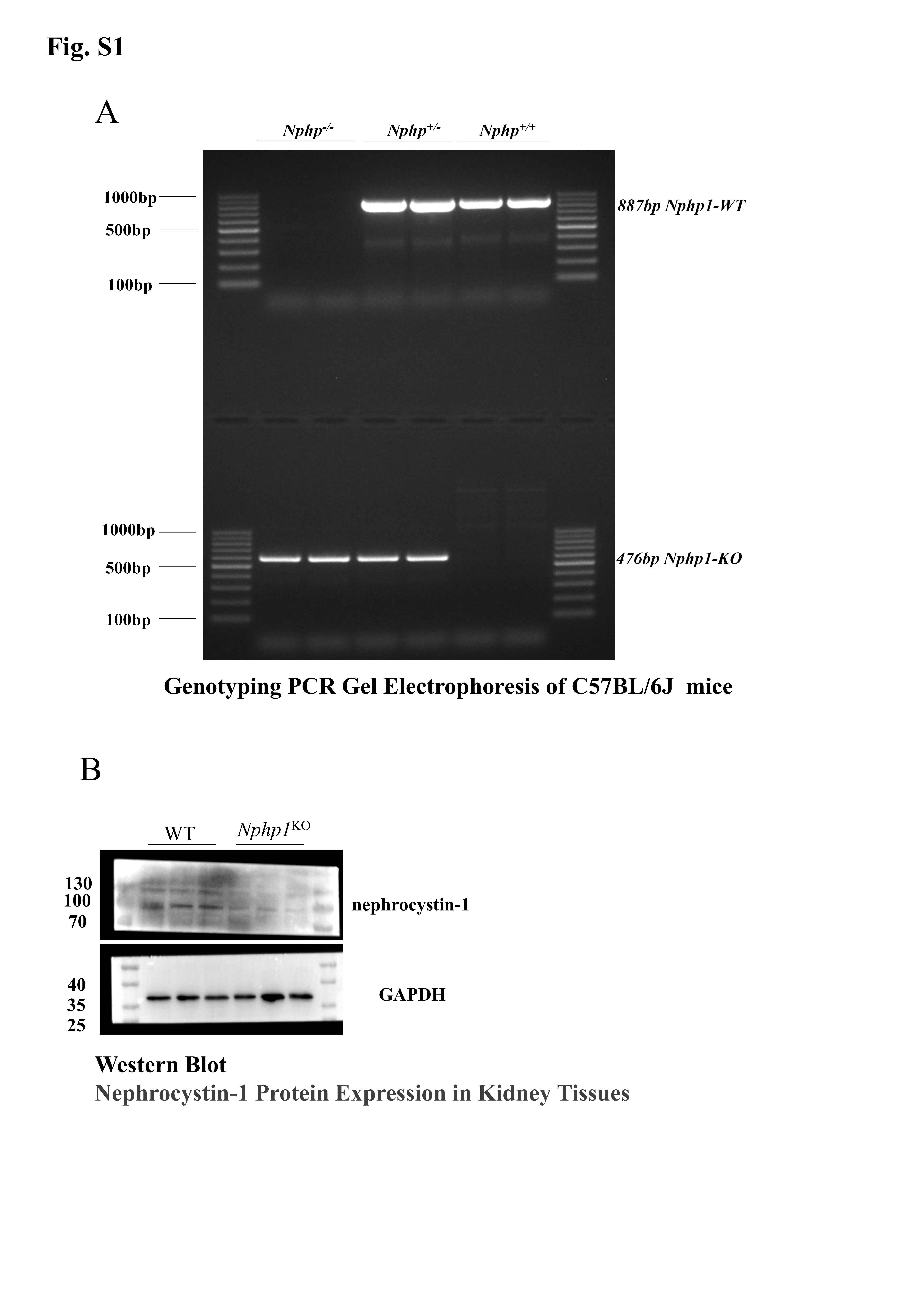

Supplement: Fig S1.tif [file IRNF_A_2684840_SM1090.tif]
